# Antifungal Activities of 4″,6″-Disubstituted Amphiphilic Kanamycins

**DOI:** 10.3390/molecules24101882

**Published:** 2019-05-16

**Authors:** Madher N. Alfindee, Yagya P. Subedi, Michelle M. Grilley, Jon Y. Takemoto, Cheng-Wei T. Chang

**Affiliations:** 1 Department of Chemistry and Biochemistry, Utah State University, 0300 Old Main Hill, Logan, UT 84322-0300, USA; madher@hotmail.com (M.N.A.); yagya.subedi@aggiemail.usu.edu (Y.P.S.); 2 Department of Biology, Utah State University, 5305 Old Main Hill, Logan, UT 84322-5305, USA; Michelle.Grilley@usu.edu (M.M.G.); jon.takemoto@usu.edu (J.Y.T.)

**Keywords:** amphiphilic kanamycin, *Cryptococcus neoformans*, antifungal, kinetic membrane permeabilization, SYTOX^TM^ green, propidium iodide

## Abstract

Amphiphilic kanamycins derived from the classic antibiotic kanamycin have attracted interest due to their novel bioactivities beyond inhibition of bacteria. In this study, the recently described 4″,6″-diaryl amphiphilic kanamycins reported as inhibitors of connexin were examined for their antifungal activities. Nearly all 4″,6″-diaryl amphiphilic kanamycins tested had antifungal activities comparable to those of 4″,6″-dialkyl amphiphilic kanamycins, reported previously against several fungal strains. The minimal growth inhibitory concentrations (MICs) correlated with the degree of amphiphilicity (cLogD) of the di-substituted amphiphilic kanamycins. Using the fluorogenic dyes, SYTOX^TM^ Green and propidium iodide, the most active compounds at the corresponding MICs or at 2×MICs caused biphasic dye fluorescence increases over time with intact cells. Further lowering the concentrations to half MICs caused first-order dye fluorescence increases. Interestingly, 4×MIC or 8×MIC levels resulted in fluorescence suppression that did not correlate with the MIC and plasma membrane permeabilization. The results show that 4″,6″-diaryl amphiphilic kanamycins are antifungal and that amphiphilicity parameter cLogD is useful for the design of the most membrane-active versions. A cautionary limitation of fluorescence suppression was revealed when using fluorogenic dyes to measure cell-permeation mechanisms with these antifungals at high concentrations. Finally, 4″,6″-diaryl amphiphilic kanamycins elevate the production of cellular reactive oxygen species as other reported amphiphilic kanamycins.

## 1. Introduction

Although commonly unappreciated, fungal diseases cause tremendous economic loss and health impacts globally. Annually, more than a billion people acquire fungal infections. The death rate from fungal diseases is equal to that of tuberculosis and malaria combined, with approximately 10% of those deaths due to cryptococcal meningitis [1,2]. Immunosuppressed individuals, such as those infected with HIV or undergoing treatment for cancer or organ transplants, are especially susceptible to invasive fungal infections. Current treatment options for fungal infections include polyene-based compounds (e.g., amphotericin B) [3,4,5], cytosine-based compounds (e.g., flucytosine) [5,6], and azole-based compounds (e.g., itraconazole and fluconazole) [5,7,8]. However, as exemplified in the recent outbreak of *Candida auris* [9], drug resistance is a growing problem. Further research to develop new and effective treatments for fungal diseases is urgent.

**Figure 1:**
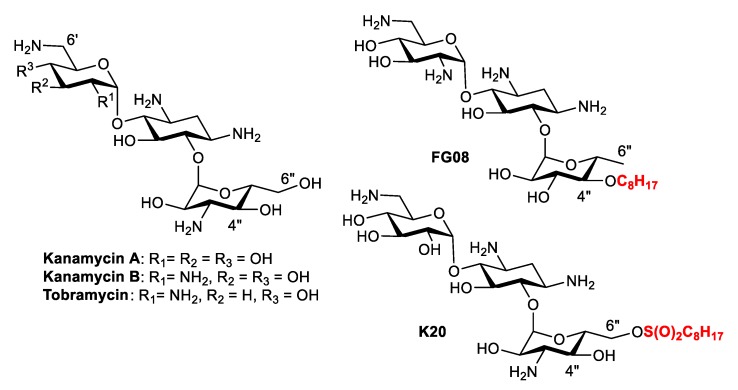
Structure of aminoglycosides and selected kanamycins (AKs).

Kanamycin belongs to a class of antibacterial compounds known as aminoglycosides that are active against both Gram-negative (G^−^) and Gram-positive (G^+^) bacteria, albeit its clinical use is limited due to the emergence of bacterial resistance (Figure 1) [10]. To overcome the problem of bacterial resistance, extensive research has been devoted to structural modifications of aminoglycosides that lead to the discovery of amphiphilic aminoglycosides [11,12]. In contrast to antibacterial kanamycin that is inactive against fungi, some of the amphiphilic kanamycins (AKs) were found to be active against a wide range of fungal strains [13,14] and concomitantly not active against bacteria. Two of the antifungal AKs produced good specific antifungal activity: **FG08**, which contains an octyl group (C8) chain attached at the 4″ position of kanamycin analog via an ether linkage [15] and **K20**, which has an octanesulfonyl group at the 6″ position (Figure 1) [16].

**Figure 2:**
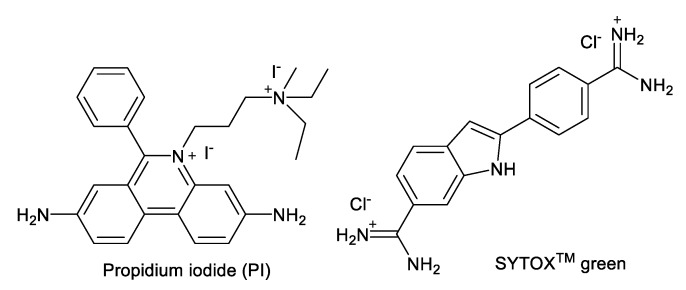
Structures of SYTOX^TM^ green and propidium iodide (PI).

**Figure 3:**
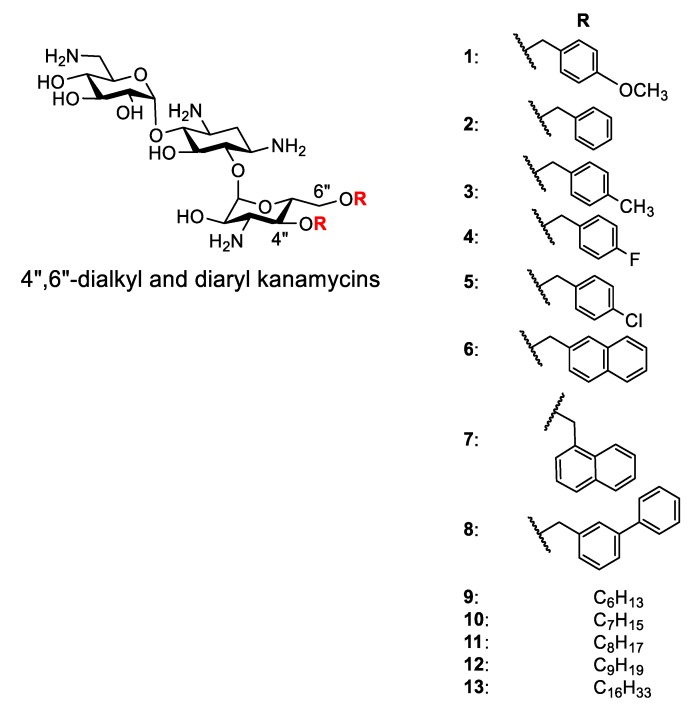
Structure of selected AKs.

AKs are known to show their antimicrobial activity by increasing the membrane permeability of microorganisms [17,18,19,20,21]. Fluorogenic dyes, such as SYTOX^TM^ green and propidium iodide (PI), are commonly employed for the study of membrane permeabilization (Figure 2). SYTOX^TM^ green is non-fluorescent and cannot penetrate the plasma membrane of intact organisms. However, in the presence of agents that compromise membrane integrity, SYTOX^TM^ green enters the cytoplasm, binds to nucleic acids, and emits fluorescence. Propidium iodide (PI) has similar properties and is widely used for evaluating membrane permeabilization of substances in fungi and bacteria. We recently reported the synthesis of 4″,6″-diaryl AKs (compounds **1**–**8**) as connexin inhibitors [22,23] (Figure 3). Since these are structurally similar to the antifungal 4″,6″-dialkyl AKs (compounds **9**–**13**), we decided to investigate their antifungal activities and to conduct mode of action studies of both groups of compounds using fluorogenic dyes.

## 2. Results and Discussion

### 2.1. Fungal Growth Inhibition by 4″,6″-Disubstituted AKs

Thirteen disubstituted AKs were examined for growth inhibition capabilities against a panel of fungi that included yeasts and the filamentous fungus *Fusarium graminearum* (Table 1). The 4″,6″- diaryl compounds **6**, **7**, and **8** were strongly inhibitory toward *F. graminearum* (minimal growth inhibitory concentrations, MICs, of 2–16 µg/mL) and compounds **1**–**5** had low (MICs, >32 µg/mL) to moderate (MICs, 16–32 µg/mL) inhibitory activities. Likewise, except with compounds **1** and **2**, *Cryptococcus neoformans* H99 and *C. neoformans* VR-54 were highly susceptible to the diaryl compounds (MICs, 2–16 µg/mL), as was *Rhodotorula pilimanae* (ATCC 26423) (MICs, 2–32 µg/mL). In contrast, except with compounds **4** and **6**, *Candida albicans* 64124 (azole-resistant) and *C. albicans* MYA2876 (azole sensitive) were not susceptible to the diaryl compounds (MICs, >32). The 4″,6″-dialkyl compounds, except **13**, displayed strong inhibitory activities against the *C. neoformans* strains (MICs, 2–16 µg/mL), and moderate activities against the *Candida* strains 64124 and MYA2876 (MICs, 8–128 µg/mL). Compound **13**, with a long linear alkyl chain (C16), had no antifungal activity (MICs, equal to or >256). Overall, except for compounds **1**, **2** and **13**, the 4″,6″-disubstituted AKs (10 of 13) were strongly inhibitory to *F. graminearum*, *C. neoformans* and *R. pilimanae* (ATCC 26423), but less so or moderately with the *C. albicans* strains.

### 2.2. Analysis of Correlation Between MIC and cLogD

**Figure 4:**
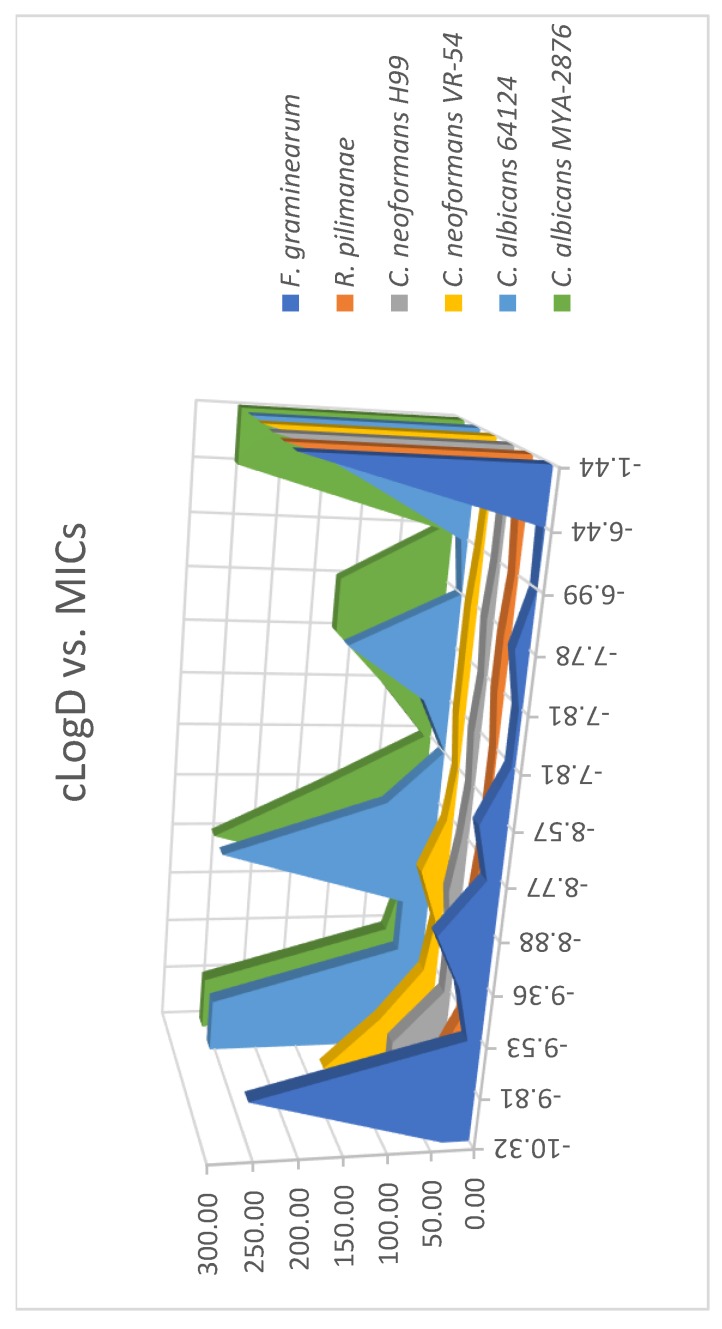
Relationship of the water/1-octanol distribution coefficient (cLogD) vs. minimal growth inhibitory concentration (MIC).

Structurally diverse AKs have been synthesized for the purpose of elucidating structure-activity relationships (SAR) [7,20]. These AKs carry variations of hydrophobic moieties or linkages of the hydrophobic moieties to the kanamycin core. Several factors that may contribute to the differences of antimicrobial activity, such as the chain length of the hydrophobic moiety and the linkage, were deduced primarily on the revealed MICs of these AKs. Despite these labor-intensive efforts, contradictory SARs were noted and these factors seemed to escalate the complexity in understanding the nature of the antimicrobial selectivity of AKs. Thus, we explored the use of a water/1-octanol distribution coefficient (cLogD) as a simplified means to evaluate the amphiphilicity of the disubstituted AK compounds and possible correlation with antifungal activity. The cLogD was calculated using Marvin Sketch (version 18.19) keeping a 0.1 molar concentration of Na^+^, K^+^, and Cl^−^ ions (Table 1). When plotting cLogD (*x*-axis) vs. MICs (*y*-axis) for all tested fungi (*z*-axis), a clearer SAR was observed (Figure 4). Compounds with cLogD values between –9.5 and –6.4 had the lowest MIC values against *F. graminearum*, *C. neoformans* (two strains) and *R. pilimanae*. cLogD values above (e.g., compound **13**) or below (e.g., compounds **1** and **2**) this range corresponded to the highest MIC values. Such a trend was not observed with *C. albicans* (two strains) because of fluctuating MIC values. This analysis suggests that it is possible to use cLogD as a guideline for designing antifungal AK of the same class, which can drastically reduce the synthetic burden.

### 2.3. Plasma Membrane Permeabilization by 4″,6″-Disubstituted Kanamycins

**Figure 5:**
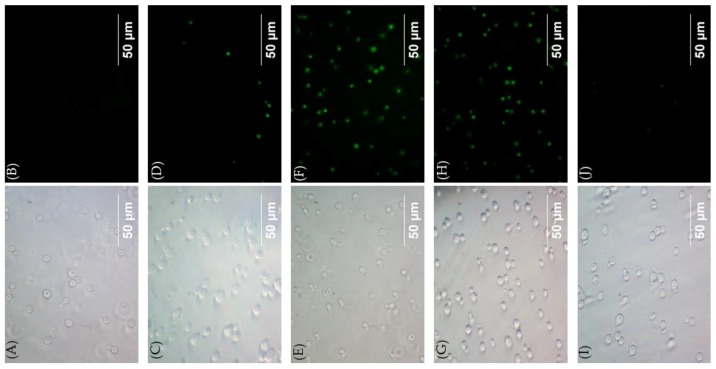
Phase contrast (left panels ) and fluorescent (right panels) images of *C. neoformans* H99. (**A**) and (**B**): Images of cells treated with SYTOX^TM^ green alone; (**C**) and (**D**): Images of cells treated with SYTOX^TM^ green and 1% Triton X-100; (**E**) and (**F**): Images of cells treated with SYTOX^TM^ green and compound **7** (1×MIC); (**G**) and (**H**): Images of cells treated with SYTOX^TM^ green and compound **11** (1xMIC); (**I**) and (**J**): Images of cells treated with SYTOX^TM^ green and compound **13** (256 μg/mL).

Plasma membrane permeabilization studies were conducted by light microscopy using the dyes SYTOX^TM^ green with *C. neoformans* H99 treated with diaryl compound **7** or the dialkyl compounds (**11** and **13**) at 1×MIC (Figure 5). Triton X-100 (1%), a non-fungal targeting agent, but known to cause membrane permeabilization of mammalian cells, was used for comparison. As expected, most fungal cells treated with compounds **7** and **11** showed fluorescence emitted from SYTOX^TM^ green. In contrast, almost no cells emitted fluorescence when treated with compound **13** (256 μg/mL). A few fungal cells emitted fluorescence when treated with Triton X-100, suggesting that this agent at 1% concentration causes a small degree of membrane permeabilization.

We previously reported that fungi treated with active AKs with hydrophobic groups attached at the 6′ position displayed a fast increase of dye fluorescence observed in a time-dependent fashion [24]. To determine if the disubstituted AKs behave similarly, time-dependent kinetic membrane permeabilization experiments were performed using *C. neoformans* H99 fungi at 1×MIC of the AKs. The relative fluorescence unit (RFU) was monitored every 3 min for 4 h.

**Figure 6:**
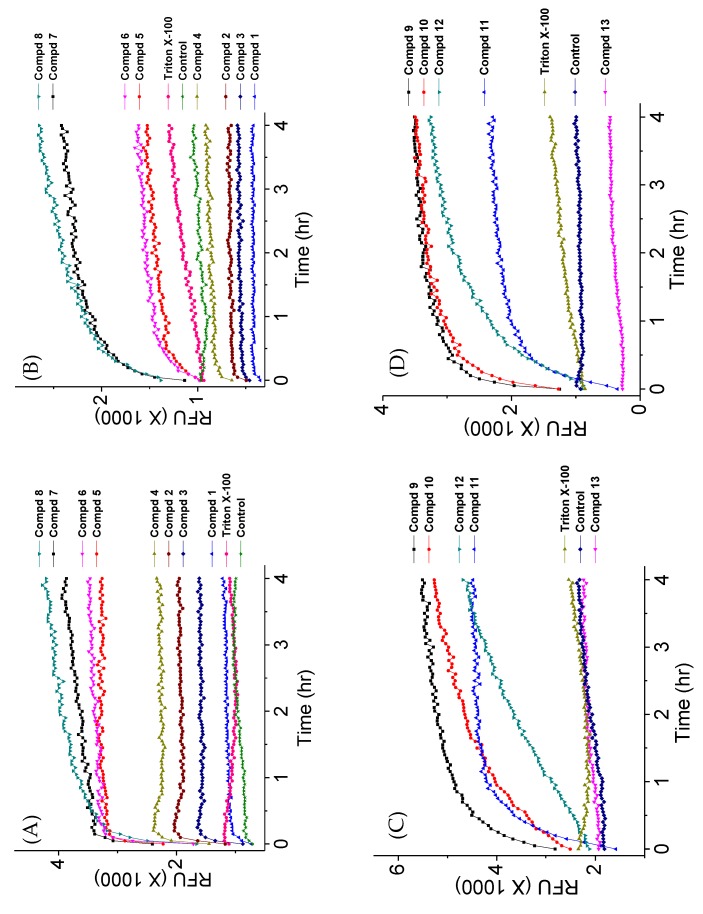
Kinetic membrane permeabilization of *C. neoformans* H99. (**A**) Cells treated with 4″,6″-diaryl AKs using SYTOX^TM^ green; (**B**) cells treated with 4″,6″-diaryl AKs using PI; (**C**) cells treated with 4″,6″-dialkyl AKs using SYTOX^TM^ green; (**D**) cells treated with 4″,6″-dialkyl AKs using PI. The figure with standard deviation is available in Supplementary Materials.

The most growth inhibitory 4″,6″-disubstituted AKs (compounds **5**, **6**, **7**, and **8**) caused the highest levels of SYTOX^TM^ green fluorescence. For most of the tested compounds, the kinetics of the fluorescence signals revealed biphasic fluorescence increases over time: A fast membrane permeabilization within the first 15 min that quickly levels to various RFUs (Figure 6). The leveled units, in general, follow similar orders in all experiments using SYTOX^TM^ green or PI. The less active AKs (**1**, **2** and **3**) showed profiles similar to Triton X-100. Despite the overall similarities of the biphasic fluorescence profiles, differences were observed between the profiles. First, different total RFU levels (at 4 h) were achieved with different AKs and with the two dyes despite using the same fungal cell densities, fluorogenic dye concentrations, and compounds at 1×MIC. Second, in three experiments (Figure 6B–D), several AKs caused total RFU levels lower than controls with no AK. Third, certain compounds displayed different RFU kinetic profiles using SYTOX^TM^ green vs. PI. For example, the RFU kinetic profiles of compounds **2** and **4** appeared flat or linear using PI (Figure 6B) but biphasic using SYTOX^TM^ green (Figure 6A). For compounds **5** and **6**, different RFU profiles were obtained from the experiments using SYTOX^TM^ green vs. PI. For compound **12**, the RFU profiles were linear using SYTOX^TM^ green but biphasic using PI (Figure 6C,D). Because the only variable parameters in these experiments were the individual AKs and their MICs, it was speculated that the fluorescence properties of the dyes reflected in the RFU and kinetic profiles were directly affected by individual AKs and their concentrations. To explore this hypothesis, we carried out further experiments using 2-fold adjusted concentrations of the MICs of the AKs.

**Figure 7:**
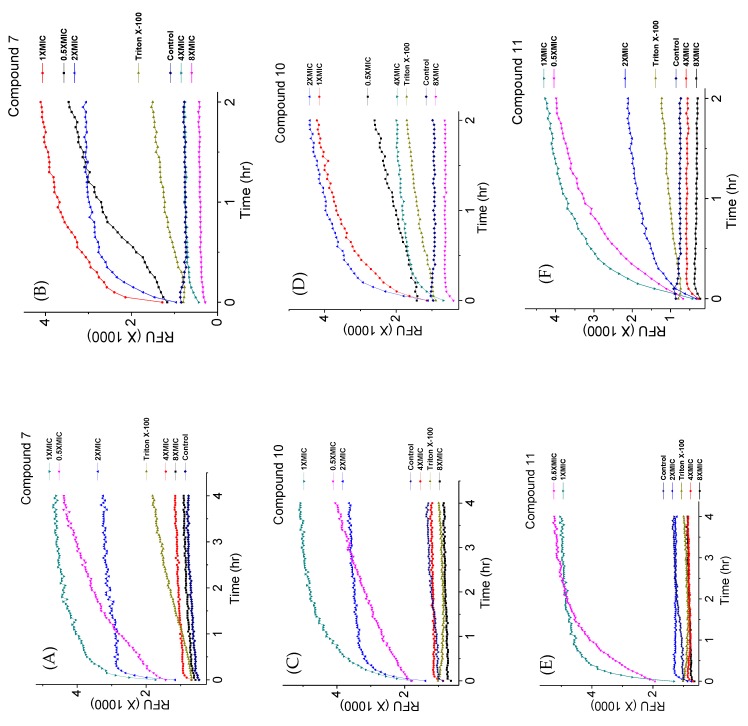
Kinetic membrane permeabilization of *C. neoformans* H99 using varied concentrations of AKs. (**A**) Cells treated with compound **7** using SYTOX^TM^ green; (**B**) cells treated with compound **7** using PI; (**C** cells treated with compound **10** using SYTOX^TM^ green; (**D**): cells treated with compound **10** using PI; (**E** cells treated with compound **11** using SYTOX^TM^ green; (**F**): cells treated with compound **11** using PI. The figure with standard deviation is available in Supplementary Materials.

Fluorescence kinetics and levels with selected 4″,6″-disubstituted AKs at various concentrations were measured with *C. neoformans* H99 using SYTOX^TM^ green and PI (Figure 7). It was observed that the AK concentration significantly influenced the degree of fluorescence independently of the MIC and membrane permeabilization capabilities. For example, compound **7** showed linear fluorescence kinetics at 0.5×MIC, biphasic kinetics at 1×MIC, biphasic kinetics but leveled at lower RFUs at 2×MIC, and suppression at 4×MIC and 8×MIC (Figure 7A,B). The same trend was observed when using PI. For compound **10**, similar kinetic profiles were obtained at 1×MIC but not when 2×MIC was employed (Figure 7C,D). The kinetic RFU profiles of compound **11** behaved like those of compound **7** (Figure 7E,F). These data support our speculation that the fluorescence of dyes can be affected by the concentration of the tested AK compounds.

**Figure 8:**
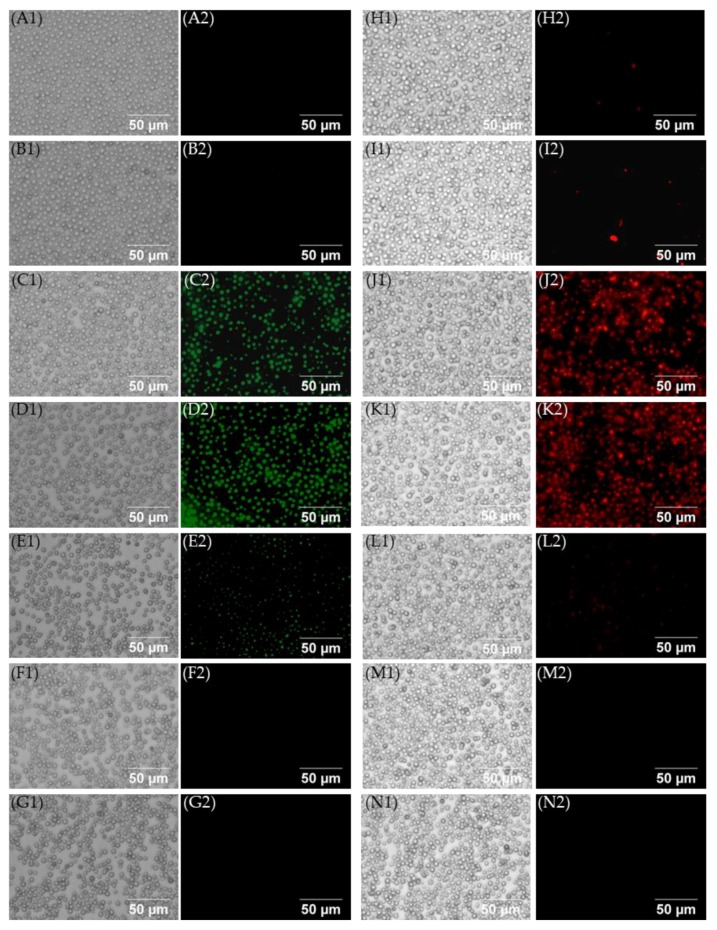
Images of *C. neoformans* treated with varied concentrations of compound **11**. (**A1,A2**) Cells treated with only SYTOX^TM^ green; (**B1,B2**) cells treated with Triton X-100 and SYTOX^TM^ green; (**C1,C2**) cells treated with 0.5×MIC of **11** and SYTOX^TM^ green; (**D1,D2**) cells treated with 1×MIC of **11** and SYTOX^TM^ green; (**E1,E2**) cells treated with 2×MIC of **11** and SYTOX^TM^ green; (**F1,F2**) cells treated with 4×MIC of **11** and SYTOX^TM^ green; (**G1,G2**) cells treated with 8×MIC of **11** and SYTOX^TM^ green; (**H1,H2**) cells treated with only PI; (**I1,I2**) cells treated with Triton X-100 and PI; (**J1,J2**) cells treated with 0.5×MIC of **11** and PI; (**K1,K2**) cells treated with 1×MIC of **11** and PI; (**L1,L2**) cells treated with 2×MIC of **11** and PI; (**M1,M2**) cells treated with 4×MIC of **11** and PI; (**N1,N2**) cells treated with 8×MIC of **11** and PI.

Images of fungi treated with compound **11** provide further evidence for the direct effect of the AK concentration on fluorescence (Figure 8). Cells treated with compound **11** and PI for 2 h emit fluorescence at the 0.5× and 1×MIC×MICs but little or no fluorescence at 8×MIC. A similar result was obtained with SYTOX^TM^ green.

These results indicate that there is an optimal ratio of AK vs. fluorogenic dye when measuring plasma membrane permeabilization by AK compounds. By considering the MICs that gave rise to the maximum leveled RFU, the concentration fell in the region around 5 µM of AK and 0.01 µM of SYTOX^TM^ green or 0.4 µM of PI. Judging from the structures of the AKs, SYTOX^TM^ green and PI (Figure 2), it is likely that ionic or hydrogen bonding contributed to the AK inhibiting effect toward these fluorogenic dyes. Therefore, caution needs to be taken when evaluating the cellular effect of AKs or other classes of compounds using fluorogenic probes. Finally, since high RFUs were observed with the use of AK levels at <1×MIC, possibilities are opened for using such low AK concentrations to detect fungi without significant fungicidal effect.

### 2.4. Effect of 4″,6″-Disubstituted Kanamycins on the Production of Reactive Oxygen Species

**Figure 9:**
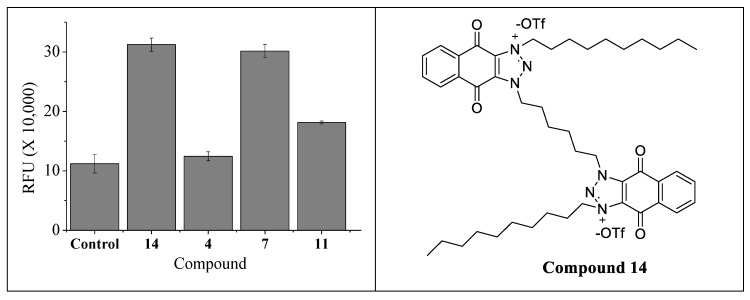
Reactive oxygen species (ROS) study for 4″,6″-disubstituted AKs and compound 14 structure.

It has been reported that a wide range of antimicrobials of different classes, albeit having various modes of action, will have a common effect of increased oxidative stress by promoting the production of reactive oxygen species (ROS), which can lead to cell death. We have also demonstrated that 6′-substituted AKs exert this common effect. Thus, several selective 4″,6″-disubstituted kanamycins—compounds **4**, **7** and **11**—were subjected to the investigation of induced ROS production. The experiments of ROS production were conducted using 1,1′-(hexane-1,6-diyl)bis(3-decyl-4,9-dioxo-4,9-dihydro-1H-naphtho [2,3-d][1,2,3]triazol-3-ium triflate (**14**) (Figure 9) [25,26], known to produce ROS, as the positive control. Compound **4** showed almost no ROS generation as compared to control, while compound **11** showed moderate ROS generation, and compound **7** showed a much higher level of ROS generation (Figure 9).

Compound **7** contains a naphthalene motif that can better stabilize radicals. Hence, it explains why compound **7** promoted ROS production to the level of the positive control, compound **14**. Compound **11**, with the linear alkyl chain, exerted a moderate level of ROS production similar to the 6′-alkylated AKs, as we have noted previously. Fluoro-substituted molecules, such as in the case of compound **4**, are noted to inhibit the formation of radicals [27]. Therefore, it is not surprising that compound **4** displayed almost no elevation of ROS production, as compared to the control (blank). However, compound **4** showed similar antifungal activities as compounds **7** and **11**. The ROS measurement was conducted following 3 h of incubation of fungi with the AKs, a much shorter time compared to the determination of the MIC, which often requires 48 h of incubation. Thus, compound **4** may exert fast membrane permeabilization, as observed with the fast RFU increases. However, the presence of fluoro-substituents deters the formation of ROS, resulting in the lower ROS generation but similar antifungal activity. Hence, it is likely that the actual mode of the antifungal mode of action is a combination of two main factors: (1) The rate of membrane permeabilization and (2) the rate of ROS production.

## 3. Materials and Methods

**Procedure for the antifungal activity.** The fungal MIC test was carried out similar to previously reported protocols [28]. In brief, fungi were grown in RPMI 1640 medium for 48 h at 37 °C. Cells, or spores in the case of *F. graminearum*, were counted and diluted to 4 × 10^4^ cells/mL in the growth medium. Fungi were added to the compounds dissolved in the growth medium, maintaining final concentrations of the compounds from 256 to 0.125 µg/mL. Fungi, treated with compounds, were incubated at 37 °C for 48 h to see the inhibitory effect of the compounds. The fungal MIC testing was done in triplicate trials.

**Procedure for kinetic cell permeabilization using 1×MIC of compounds.** The fungi cell membrane permeabilization study was performed in a 96-well cell culture plate. *C. neoformans* H99 was grown in PDB medium at 37 °C for 48 h. From the growth, 4 mL of fungi were taken and washed with water twice and resuspended in 1 mL of water. Fungi cells were counted, and cells were diluted to 4 × 10^6^ cells/mL as the final confluence. Then, compounds and dye were added to the fungi, maintaining the final concentration of compounds to 1×MIC, 0.01 µM for SYTOX^TM^ green and 0.4 µg/mL for PI. The control cells were treated with dye only. The fluorescence intensity was measured in every 3 min for a period of four hours at 28 °C with an excitation/emission wavelength of 480_Ex_/525_Em_ nm for SYTOX^TM^ green and 537_Ex_/617_Em_ nm for PI using the Cytation 5 imaging reader. The images of compounds **7**, **11**, **13**, and Triton X-100 were taken after 60 min of incubation using the green channel and bright field filter set in the Cytation 5 imaging reader and Olympus IX 71.

**Procedure for kinetic cell permeabilization using multiple MIC of the compounds.** This assay was performed similarly to the fungi cell membrane permeabilization study using 0.125×MIC to 8×MIC of compounds **2**, **7**, **10**, and **11**. The fluorescence intensity was measured every 3 min for a period of two or four hours. The images of the representative compound **11** were taken after the 60 min of incubation using the green channel for SYTOX^TM^ green and after 120 min using the red channel for PI along with the bright field filter set.

**Procedure for reactive oxygen species (ROS) generation study.** The effect of the amphiphilic kanamycin to generate ROS species in the fungal cells was studied in the *C. neoformans* H99 strain, using the previously reported ROS-generating amphiphilic antifungal compound **14**, as the positive control [25,26]. The fungal cells were grown in PDB medium at 28 °C for 48 h with gentle shaking. The cells were washed with water twice before using for the assay by centrifugation at 10,000 rpm for 2 min. A volume of 0.5 mL of cells in water (final cell confluence, 4 × 10^7^ cells/mL) was incubated with 1×MIC of the compounds for 3 h at 37 °C. Then, the cells were washed twice with 0.5 mL water and finally resuspended in 0.5 mL water. The cells were then incubated for another 30 min with 2′,7′-dichlorodihydrofluorescein diacetate (DCF-DA) dye (25 mM final concentration) at 37 °C. After incubation, the cells were washed twice with the same volume of water and resuspended in water. The fluorescence of the cells was measured in the Cytation 5 imaging reader with an excitation and emission wavelength of 485 nm and 525 nm, respectively. The experiment was performed at least in triplicate.

## 4. Conclusions

We have examined the antifungal activities of 4″,6″-diaryl kanamycins, as well as the previously described 4″,6″-dialkyl kanamycins. These two classes of AKs show similar antifungal profiles against a panel of fungi despite having different hydrophobic groups. The amphiphilicity parameter cLogD can be useful for the structural design when no obvious SAR can be deduced. Using the fluorogenic dye SYTOX^TM^ green—4″,6″-diaryl kanamycins were shown to exert their antifungal activities via the increase of plasma membrane permeability. It was shown, however, that the fluorescent properties of the commonly used fluorogenic dyes SYTOX^TM^ green and PI are drastically affected by the concentrations of the AKs. Therefore, to acquire meaningful results in such approaches, prudent practice is needed to establish the optimal ratio of AKs to fluorogenic dyes for the experiments. Finally, from a combination of studying kinetic membrane permeabilization, ROS production, cLogD and fungal growth MICs, we conclude that the mode of antifungal action of the 4″,6″-disubstituted AKs is a combination of plasma membrane permeabilization and oxidative stress.

## Figures and Tables

**Table 1:** Minimal growth inhibitory concentrations (MICs) of AKs against fungal strains.

Compound	cLogD	Strains					
		A	B	C	D	E	F
**1**	−10.32	32–64	256	256	32	64	128
**2**	−9.81	256	256	>256	32	64	64
**3**	−8.88	64–128	>256	256	8	16	32
**4**	−9.53	16–32	32–64	32–64	4	8	16
**5**	−8.77	16–32	64	128	4–8	8	8
**6**	−7.81	4–16	32–64	64	2–4	8	8
**7**	−7.81	4−8	128	128	8–16	4	8
**8**	−6.44	2–8	128–256	256	8–16	4	2–4
**9**	−9.36	32	32	16–32	8–16	8	8
**10**	−8.57	32	ND	8–16	2	4	2
**11**	−7.78	16	ND	128	8–16	8	8–16
**12**	−6.99	ND	8	16–32	4	4	4
**13**	−1.44	>256	>256	>256	>256	256	>256
**K20**	−11.62	32	16	32	8–16	8	16

MIC unit: µg/mL; ND: Not determined; strains: (**A**) *Fusarium graminearum* B4-5A, (**B**) *Candida albicans* 64124, (**C**) *Candida albicans* MYA-2876, (**D**) *Rhodotorula pilimanae* (ATCC 26423), (**E**) *Cryptococcus neoformans* H99, (**F**) *Cryptococcus neoformans* VR-54.

## Supplementary Materials

Graphs of the kinetic membrane permeabilization of *C. neoformans* with 1×MIC of compounds with the standard deviation and graphs of the kinetic membrane permeabilization of *C. neoformans* with multiple MICs of compounds with standard deviation are available online.
