# Supplementary material for: Antifungal Activities of 4″,6″-Disubstituted Amphiphilic Kanamycins
Source: Molecules. 2019 May 16;24(10):1882. doi: 10.3390/molecules24101882 (PMC6571828; doi:10.3390/molecules24101882)
Supplement: Supplementary file 1 [file molecules-24-01882-s001.pdf]

molecules

MDPI

Supplementary material

Study of the Antifungal Property of 4′′,6′′-Disubstituted Amphiphilic Kanamycins

Madher N. AlFindee [1]***, Yagya P. Subedi*** [1]***, Michelle M. Grilley*** [2]***, Jon Y. Takemoto*** [2] ***and Cheng-Wei T. Chang*** [1,*]

[1] Department of Chemistry and Biochemistry, Utah State University, 0300 Old Main Hill, Logan, Utah 84322-0300, USA

[2] Department of Biology, Utah State University, 5305 Old Main Hill, Logan, Utah 84322-5305, USA

* Correspondence: tom.chang@usu.edu

Table of contents **Pages**

1. Kinetic membrane permeabilization of *C. neoformans* with 1xMIC of compounds S2
2. Kinetic membrane permeabilization of *C. neoformans* with multiple MIC of Compounds S2 - S3

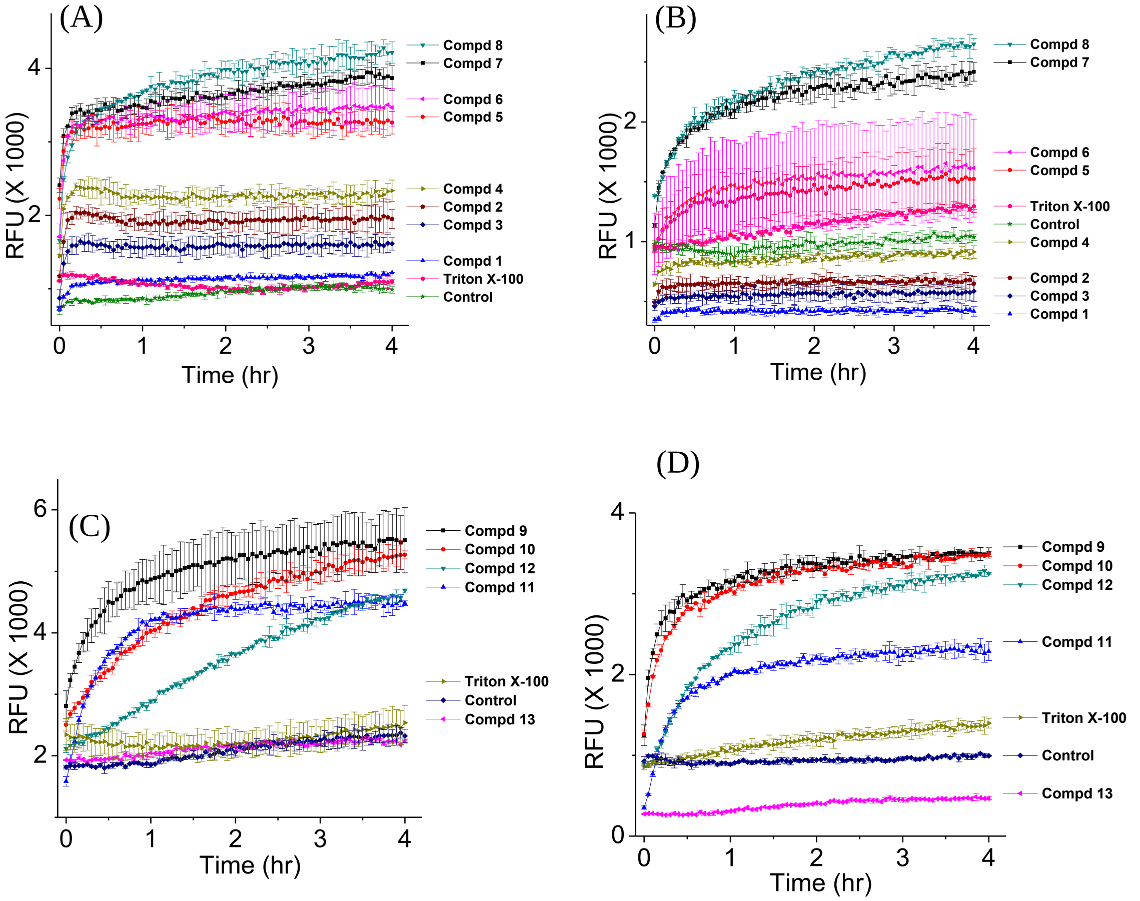

Figure S1. Kinetic Membrane Permeabilization of *C. neoformans* treated with: (A) AKs attached with aryl groups and Sytox; (B) AKs attached with aryl groups and PI; (C) AKs attached with alkyl groups and Sytox; (D) AKs attached with alkyl groups and PI.

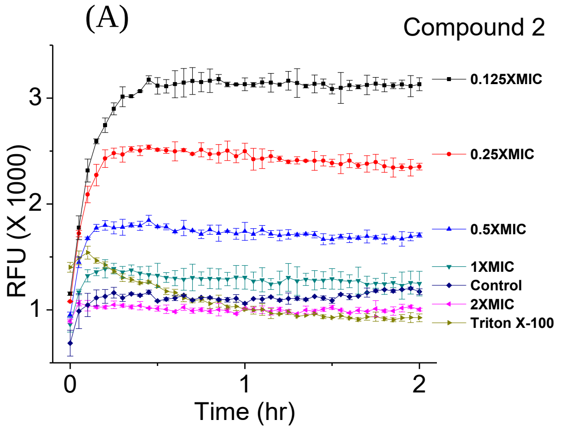

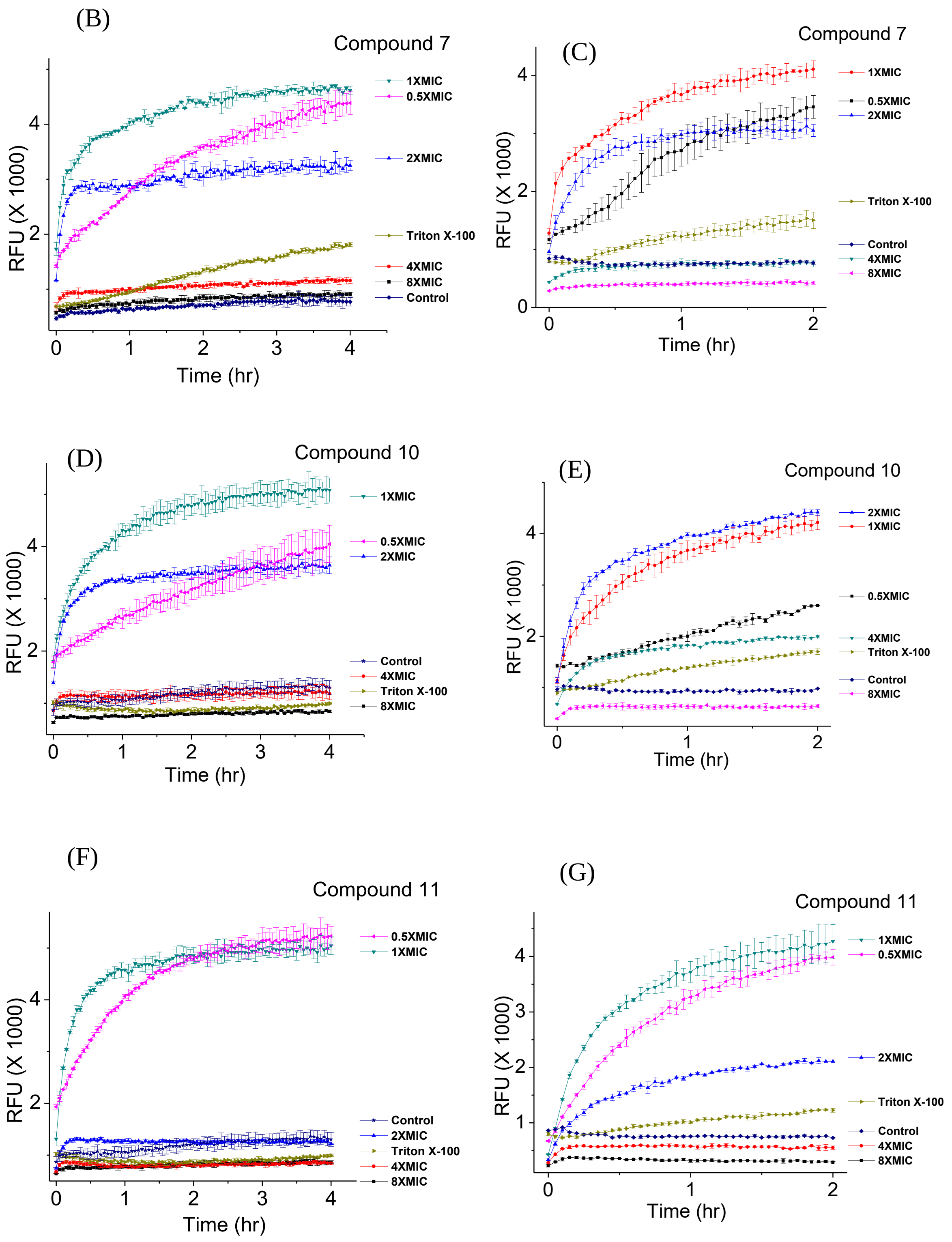

Figure S2. Kinetic membrane permeabilization of *C. neoformans* treated with multiple MIC of : (A) **2** and 0.01 µM Sytox; (B) **7** and 0.01 µM Sytox; (C)) **7** and and 0.4 µg/mL PI; (D)) **10** and 0.01 µM Sytox; (E) **10** and 0.4 µg/mL PI; (F) **11 and** 0.01 µM Sytox ; (G) **11** and 0.4 µg/mL PI.
